# Supplementary material for: Content-rich biological network constructed by mining PubMed abstracts
Source: BMC Bioinformatics. 2004 Oct 8;5:147. doi: 10.1186/1471-2105-5-147 (PMC528731; doi:10.1186/1471-2105-5-147)
Supplement: Additional File 2 — The original results of the above study (non-essential files are deleted to keep the file size under the limit set by BMC bioinformatics). [file 1471-2105-5-147-S2.bz2 › chilibotAdditionalFile2/dip20/228ID7954478E745/html/CTNNB1_CDH1.html]

 


 **CTNNB1** and **CDH1** 
  
Found 952 abstracts in PubMed, retrieved 20.  
 

 What does Google say? 
 PDF only 
| .edu only 

---

**Interactive relationship** (e.g. stimulation, inhibition, etc)

**Inhibitory relationship**- Reduced expression of E cadherin  [ **CDH1** ] , which interacts with beta catenin  [ **CTNNB1** ]  at the membrane, was observed in some tumors, but this did not correlate with Catnb mutation.  Ref: 12878043 Toxicol Appl Pharmacol, 2003
- However, alteration in the beta catenin  [ **CTNNB1** ]  TCF transcriptional regulation of target genes is not required for the invasion suppressor activity of E cadherin  [ **CDH1** ] , suggesting the involvement of other beta catenin  [ **CTNNB1** ]  binding proteins.  Ref: 12810698 J Cell Biol, 2003
- In conclusion our results suggested that beta catenin  [ **CTNNB1** ]  alteration might be a minor contributor to the development of gallbladder carcinomas through abnormal Wnt wingless signalling decreased membranous expression of beta catenin  [ **CTNNB1** ]  might be correlated to carcinoma progression through loss of cell adhesive function in E cadherin  [ **CDH1** ]  catenin  [ **CTNNB1** ]  fashion.  Ref: 12866584 J Exp Clin Cancer Res, 2003
- The general pattern of gene expression in IC and APF treated cells suggested a less proliferative phenotype, with increased expression of E cadherin  [ **CDH1** ] , phosphoribosylpyrophosphate synthetase associated protein 39, and SWI SNF complex 170 kDa subunit, and decreased expression of vimentin, alpha2 integrin, alpha1 catenin  [ **CTNNB1** ] , cyclin D1, and jun N terminal kinase 1.  Ref: 12847144 Physiol Genomics, 2003
**Neutral relationship**- At the invading front of cancers, the E cadherin  [ **CDH1** ]  cell adhesion system is inactivated by tyrosine phosphorylation of beta catenin  [ **CTNNB1** ] .  Ref: 12841864 Cancer Sci, 2003
- As shown by immunoprecipitation, the re expressed E cadherin  [ **CDH1** ]  was able to sequestrate one part of free cytoplasmic beta catenin  [ **CTNNB1** ]  in BZR cells.  Ref: 12875984 Am J Pathol, 2003

**Non-interactive relationship** (e.g. studied together, co-existance, homology, etc.)

- Expression of E cadherin  [ **CDH1** ] , alpha catenin  [ **CTNNB1** ] , and beta catenin  [ **CTNNB1** ]  in the process of lymph node metastasis in oral squamous cell carcinoma.  Ref: 12888830 Br J Cancer, 2003
- To test this hypothesis, we have analyzed the presence of genetic and epigenetic alterations affecting E CD  **CDH1** , APC and beta catenin  [ **CTNNB1** ]   **CTNNB1**  genes and the immunohistochemical expression of E CD, beta and gamma catenin  [ **CTNNB1** ]  in a series of 46 infiltrating lobular breast carcinomas ILCs.  Ref: 12800196 Int J Cancer, 2003
- Epigenetic and genetic alterations of APC and  **CDH1**  genes in lobular breast cancer Relationships with abnormal E cadherin  [ **CDH1** ]  and catenin  [ **CTNNB1** ]  expression and microsatellite instability.  Ref: 12800196 Int J Cancer, 2003
- Rather, the invasion suppressor signal is mediated through the beta catenin  [ **CTNNB1** ]  binding domain of the E cadherin  [ **CDH1** ]  cytoplasmic tail but not through the p120ctn binding domain.  Ref: 12810698 J Cell Biol, 2003
- We observed immunoreactivity for APC 147 kD, beta catenin  [ **CTNNB1** ]  92 kD, E cadherin  [ **CDH1** ]  120 and 84 kD and occludin 70 85 kD by western blotting.  Ref: 12850834 Platelets, 2003
- Western blotting was used to determine polypeptide size and expression of E cadherin  [ **CDH1** ]  and beta catenin  [ **CTNNB1** ]  in Wilms s tumours compared with normal kidney.  Ref: 12890743 Mol Pathol, 2003
- BACKGROUND The aim of this study was to examine the expression of E cadherin  [ **CDH1** ] , beta catenin  [ **CTNNB1** ] , CD44s and CD44v6 in gastric adenocarcinoma and to investigate whether these proteins are objective in markers in predicting the lymph node metastasis.  Ref: 12820426 Anticancer Res< MedlineTA>Anticancer Res, 2003
- These data demonstrate a direct role of E cadherin  [ **CDH1** ]  catenin  [ **CTNNB1** ]  complex organization in the regulation of MMPs and suggest an implication of this regulation in the expression of an invasive phenotype by bronchial tumor cells.  Ref: 12875984 Am J Pathol, 2003
- Distribution of cell cell adhesion molecules of E cadherin  [ **CDH1** ]  EC and beta catenin  [ **CTNNB1** ]  BC infiltrating breast carcinoma BC and markers of epithelium, basal membrane has been studied by immunofluorescence in 50 infiltrating BC tissue samples including 17 ductal carcinomas, 23 lobular carcinomas and 10 combined carcinomas.  Ref: 12879601 Arkh PatolArkh Patol, 2003
- The disorganization of E cadherin  [ **CDH1** ]  catenin  [ **CTNNB1** ]  complexes and the overexpression of matrix metalloproteinases MMPs are frequently involved in the capacity of epithelial cells to acquire an invasive phenotype.  Ref: 12875984 Am J Pathol, 2003
- We have studied the effects of the organophosphorous pesticide malathion on cell viability, actin cytoskeleton, cell adhesion complex E cadherin  [ **CDH1** ]  beta catenin  [ **CTNNB1** ] , and Rho and Rac1 GTPases from the human mammary carcinoma cell line MCF 7.  Ref: 12888906 Int J Oncol, 2003
- CONCLUSIONS Compared with primary tumours, metastatic tumours showed lower expression of E cadherin  [ **CDH1** ]  and gamma catenin  [ **CTNNB1** ] , with nuclear staining for beta catenin  [ **CTNNB1** ] .  Ref: 12890743 Mol Pathol, 2003
- RESULTS The expressions of E cadherin  [ **CDH1** ]  and beta catenin  [ **CTNNB1** ]  were significantly associated with histological type, but not with tumor progression and lymph node metastasis.  Ref: 12820426 Anticancer Res< MedlineTA>Anticancer Res, 2003
- The decrease of beta catenin  [ **CTNNB1** ]  transcriptional activity in E cadherin  [ **CDH1** ]  transfected clones was demonstrated using the TOP FLASH reporter construct.  Ref: 12875984 Am J Pathol, 2003
- METHODS Immunohistochemistry was used to determine the expression and cellular distribution of E cadherin  [ **CDH1** ] , beta catenin  [ **CTNNB1** ] , gamma catenin  [ **CTNNB1** ] , and ezrin in primary and metastatic Wilms s tumours.  Ref: 12890743 Mol Pathol, 2003
- These data suggest that evaluation of the immunohistochemical expression of E cadherin  [ **CDH1** ] , alpha catenin  [ **CTNNB1** ]  and beta catenin  [ **CTNNB1** ]  is extremely valuable for the diagnosis of metastatic occurrence.British Journal of Cancer 2003 8 57 563.  Ref: 12888830 Br J Cancer, 2003
- In the metastatic process, reduction in cell to cell adhesion including E cadherin  [ **CDH1** ]  catenin  [ **CTNNB1** ]  cell adhesion complex is an essential step.  Ref: 12888830 Br J Cancer, 2003
- However, beta and gamma catenin  [ **CTNNB1** ]  were severely reduced or absent in 90% of these tumors, implying that alterations in  **CDH1**  and APC genes do not promote beta catenin  [ **CTNNB1** ]  accumulation in ILC.  Ref: 12800196 Int J Cancer, 2003
- The E cadherin  [ **CDH1** ]  cell adhesion system cross talks with the Wingless Wnt signaling pathway through beta catenin  [ **CTNNB1** ] , and expression of genes, which participate in cancer morphogenesis, may be regulated in conjunction with the Wingless Wnt signaling pathway.  Ref: 12841864 Cancer Sci, 2003
- Influence of E cadherin  [ **CDH1** ]  promoter methylation and mutation of beta catenin  [ **CTNNB1** ]  on invasion and metastasis of nasopharyngeal carcinoma cells < ArticleTitle>.  Ref: 12839684 Zhonghua Zhong Liu Za Zhi, 2003
- Significantly greater reduction in expression levels of E cadherin  [ **CDH1** ] , alpha catenin  [ **CTNNB1** ]  and beta catenin  [ **CTNNB1** ]  was found in the metastatic group n=64 compared to the nonmetastatic group n=95 P=0.00.  Ref: 12888830 Br J Cancer, 2003
- METHODS Methylation of E cadherin  [ **CDH1** ]  promoter, mutation in exon 3 of beta catenin  [ **CTNNB1** ]  and differential expression of beta catenin  [ **CTNNB1** ]  in the primary lesion of 21 NPC and the metastatic lymphnode of 21 NPC were investigated by DNA Methylation Specific PCR, direct sequencing and immunohistochemical method.  Ref: 12839684 Zhonghua Zhong Liu Za Zhi, 2003
- especially, CD44v6 expression may be a useful predictor of lymph node metastasis, while the expressions of E cadherin  [ **CDH1** ]  and beta catenin  [ **CTNNB1** ]  complex are more probably related to tumor morphology than to tumor progression.  Ref: 12820426 Anticancer Res< MedlineTA>Anticancer Res, 2003
- Expression of potential beta catenin  [ **CTNNB1** ]  targets, cyclin D1, c Jun, c Myc, E cadherin  [ **CDH1** ] , and EGFR in chemically induced hepatocellular neoplasms from B6C3F1 mice.  Ref: 12878043 Toxicol Appl Pharmacol, 2003
- We investigated immunohistochemical expression of E cadherin  [ **CDH1** ] , alpha catenin  [ **CTNNB1** ]  and beta catenin  [ **CTNNB1** ]  in 159 tissue samples from patients with oral squamous cell carcinoma and examined the correlation between their expressions and the presence of regional lymph node metastasis.  Ref: 12888830 Br J Cancer, 2003
- Expression of E cadherin  [ **CDH1** ] , beta catenin  [ **CTNNB1** ] , CD44s and CD44v6 in gastric adenocarcinoma relationship with lymph node metastasis.  Ref: 12820426 Anticancer Res< MedlineTA>Anticancer Res, 2003
- The expression pattern of genes known to be involved in the pathogenesis of sporadic CRC was assessed immunohistochemically E cadherin  [ **CDH1** ] , beta catenin  [ **CTNNB1** ] , deleted in colon cancer DCC, and p53.  Ref: 12822869 Dig Dis Sci, 2003
- MMP 9 knockdown concomitantly resulted in increased levels of surface E cadherin  [ **CDH1** ] , redistribution at the plasma membrane of b catenin  [ **CTNNB1** ]  and its physical association with E cadherin  [ **CDH1** ] .  Ref: 12847101 J Biol Chem< MedlineTA>J Biol Chem, 2003
- AIMS To evaluate E cadherin  [ **CDH1** ] , beta catenin  [ **CTNNB1** ] , gamma catenin  [ **CTNNB1** ] , and ezrin expression and its relation to histopathological features of primary and metastatic Wilms s tumours.  Ref: 12890743 Mol Pathol, 2003
- However, altered  **CDH1**  and APC genes do not promote beta catenin  [ **CTNNB1** ]  accumulation in this tumor type.  Ref: 12800196 Int J Cancer, 2003
- Immunocharacterization with beta catenin  [ **CTNNB1** ] , E cadherin  [ **CDH1** ] , cytokeratins AE1 AE3, epithelial membrane antigen, S 100 protein, smooth muscle actin, and vimentin was also performed.  Ref: 12883237 Am J Surg Pathol, 2003
- BACKGROUND The E cadherin  [ **CDH1** ]  catenin  [ **CTNNB1** ]  adhesion complex is crucial for intercellular adhesiveness and maintenance of tissue architecture.  Ref: 12890743 Mol Pathol, 2003
- In this study, we screened liver tumors induced by 2 amino dimethylimidazo f quinoline MeIQ in CDF 1 mice for beta catenin  [ **CTNNB1** ]  and APC mutations and other genetic alterations shown to occur in human hepatocellular carcinomas HCC, including mutations in the p53 and H ras genes, c myc amplification and E cadherin  [ **CDH1** ]  promoter methylation.  Ref: 12893427 Cancer Lett, 2003
- these findings were confirmed for the structural gene products E cadherin  [ **CDH1** ] , vimentin, alpha2 integrin, and alpha catenin  [ **CTNNB1** ]  by immunohistochemistry.  Ref: 12847144 Physiol Genomics, 2003
- MATERIALS AND METHODS In 99 cases of gastric adenocarcinoma, the expressions of E cadherin  [ **CDH1** ] , beta catenin  [ **CTNNB1** ] , CD44s and CD44v6 were studied by immunohistochemistry.  Ref: 12820426 Anticancer Res< MedlineTA>Anticancer Res, 2003
- These changes were related to a decrease in the expression of the adhesion molecules, E cadherin  [ **CDH1** ]  and beta catenin  [ **CTNNB1** ] , and to the distribution and reactivity of actin microfilaments to TRITC phalloidin.  Ref: 12888906 Int J Oncol, 2003
- In this study, we investigated whether the invasion suppressor activity of E cadherin  [ **CDH1** ]  is mediated directly by tighter physical cell adhesion, indirectly by sequestering beta catenin  [ **CTNNB1** ]  and thus antagonizing beta catenin  [ **CTNNB1** ]  T cell factor TCF signaling, or by other signaling pathways.  Ref: 12810698 J Cell Biol, 2003
- Additionally, beta catenin  [ **CTNNB1** ] , pan reactive cadherins, E cadherin  [ **CDH1** ]  and occludin were seen to partition with the triton insoluble cytoskeleton in platelets.  Ref: 12850834 Platelets, 2003
- OBJECTIVE To study the mechanism of invasion and metastasis in early nasopharyngeal carcinoma NPC in relation to E cadherin  [ **CDH1** ]  promoter methylation and mutation in exon 3 of beta catenin  [ **CTNNB1** ] .  Ref: 12839684 Zhonghua Zhong Liu Za Zhi, 2003
- Tumors of I1307K mutation carriers displayed positive p53 immunostaining and loss of beta catenin  [ **CTNNB1** ] , E cadherin  [ **CDH1** ] , and DCC expression more often compared with noncarriers, although none of these differences reached statistical significance.  Ref: 12822869 Dig Dis Sci, 2003
- Differential expression of E cadherin  [ **CDH1** ]  and beta catenin  [ **CTNNB1** ]  in primary and metastatic Wilms s tumours.  Ref: 12890743 Mol Pathol, 2003
